# Directional Navigation Improves Opportunistic Communication for Emergencies

**DOI:** 10.3390/s140815387

**Published:** 2014-08-20

**Authors:** Andras. Kokuti, Erol. Gelenbe

**Affiliations:** Intelligent Systems and Networks Group, Department of Electrical and Electronic Engineering, Imperial College, London SW72AZ, UK; E-Mail: kokand@gmail.com

**Keywords:** emergency navigation, opportunistic communications, building evacuation, self organization

## Abstract

We present a novel direction based shortest path search algorithm to guide evacuees during an emergency. It uses opportunistic communications (oppcomms) with low-cost wearable mobile nodes that can exchange packets at close range of a few to some tens of meters without help of an infrastructure. The algorithm seeks the shortest path to exits which are safest with regard to a hazard, and is integrated into an autonomous Emergency Support System (ESS) to guide evacuees in a built environment. The algorithm proposed that ESSs are evaluated with the DBES (Distributed Building Evacuation Simulator) by simulating a shopping centre where fire is spreading. The results show that the directional path finding algorithm can offer significant improvements for the evacuees.

## 1. Introduction

Emergency evacuation is unfortunately an essential component of built environments such as sports arenas, concert halls, buildings in general, and even ships. The technical issues it raises are related to search techniques [1] to guide people or vehicles in dangerous areas [1,2]. As a consequence, there has been many work related to evacuation techniques that use distributed computation and wireless sensor networks (WSN) [3]. Although WSNs can be used both for sensing the hazards, identifying evacuees and communicating with them, and possibly for finding safe paths, such networks also require a minimal fixed infrastructure which remains viable during an emergency. Since many catastrophic events can also impair or destroy the fixed sensor nodes, this paper considers the use of a simpler technology based on commonly available wearable opportunistic network nodes that can be implemented with smart phones or personal digital assistants, as a supplementary system or an alternative for helping evacuees.

Research papers in the field [4] fall mainly into two categories. The first group is comprised of solutions that rely on the existence of a sensor network for emergency navigation. The work by Li *et al.* [5] who propose a distributed navigation algorithm with implementation and evaluation on a physical sensor network. Their approach is based on the flooding model in which every sensor exchanges information with every other sensor, therefore, this solution does not scale well, due to a very high communication cost. In [6] a distributed navigation algorithm based on the temporally ordered routing algorithm (TORA) is introduced in order to guide civilians in a building with a 2D layout during an emergency. This protocol is extended to 3D environments in [7]. In addition, others [8,9] have taken congestion into account, and in [8] an algorithm where rescue force flexibility is also examined is proposed. An indoor navigation system is presented in [10] based on mobile phones that use a pre-existing WSN for monitoring the building and receiving updates regarding a dynamic hazard, together with a centralized emergency guidance system. Some prior work [11,12] provides a distributed decision support system (DDSS) for building evacuation, where static decision nodes (DNs) with communication and processing capabilities are installed in the building and provide directions to civilians in their vicinity, either via dynamic signs or wireless communications. DNs form a network and disseminate information regarding the hazard and evacuation paths in a distributed manner based only on local information, and a distributed update algorithm is executed periodically by the DNs, where each DN communicates with its neighbors to send its current hazard and path metrics. Other work has considered the specific computational structure that is needed to simulate and control emergency navigation with the use of sensor networks [13].

The second group of solutions is significantly different from the first, since hazard information is disseminated over an opportunistic network of communication nodes (CNs) that operate as part of the Emergency Support System (ESS) and disseminate Emergency Messages (EM). Most data forwarding and routing protocols for opportunistic networks (oppnets) [14] seek a balance between message delivery ratio and resource consumption [15–17]. For the dissemination of EMs, which are very short in nature, a high delivery ratio and low message latencies are crucial. A fine example of opportunistic communication protocols is the epidemic routing protocol [18], which disseminates multiple copies of a message over the network similar to the spread of an infectious disease and has been used in [19].

However, the ESS we describe uses a fixed infrastructure of sensor nodes (SNs) to monitor the environment, but each civilian is guided by his/her own individual CN, which locally calculates the best evacuation path based on the currently obtained information. Thus, this paper provides an enhanced path finding algorithm to guide evacuees from the hazardous areas to safe exits in a direction that avoids hazards. This direction is estimated for each evacuee, from the current position of all the evacuees based on Opportunistic Communications (oppcomms) information sharing [19], with a directional extension of Epidemic Routing. The experimental results that we present based on simulations with the DBES tool [20], show the degree of improvement that the directional path finding algorithm can offer compared to previous approaches.

The rest of the paper is organized as follows: The novel algorithms are described in Section 2. We establish the simulation environment, perform the experiments and show the results in Section 3 and finally, the conclusions of the current work are presented in Section 4.

## 2. The Novel Algorithms

The same system was used as in [19]. This ESS consists of fixed sensor nodes (SNs) and mobile communication nodes (CNs). The sensors are pre-deployed at fixed locations in the building, having short range wireless communication capability so they can directly communicate with CNs in range. SNs are only utilized for civilian localization, *i.e.*, to tell the portable CNs about where is the mobile user in the building, as in an indoor environment GPS localization is not reliable. Therefore, SNs do not form a conventional wireless sensor network and hazard information is not disseminated among SNs, because the limited energy and physical capabilities of SNs. The communication nodes (CNs) form a network in an opportunistic manner as devices come into contact. Opportunistic communication enables the dissemination of messages in order to gather and convey information for situational awareness, such as the condition and location of the hazard. The described ESS assumes that each CN stores the graph representation of the area (*i.e.*, the layout of a shopping centre) and can communicate each other via its radio interface.

Our proposed algorithms, a path finding algorithm and a communication protocol, are based on the same idea: always to maintain the direction from the hazardous area (*i.e.*, fire area) to the exits. With the help of this direction our navigation system can guide the civilians not through the shortest path but through a safer one. Once a CN (which is carried by a civilian) observes hazard in its local environment it generates a new emergency message (EM) that includes the location of the fire (gathered from the SNs), the hazard intensity, the device ID and the observation timestamp. Each EM is identified by its (device ID, timestamp) pair. CNs use oppcomms to disseminate EMs to other CNs. From the received EMs a CN can update its own local graph (which is stored on the device) and calculates a path to the determined exit. The algorithm for the path selection will be explained in Section 2.1 while the protocol, which is used for data dissemination among CNs, will be defined in Section 2.2.

### 2.1. The Direction Based Path Finding Algorithm

**Figure 1.**
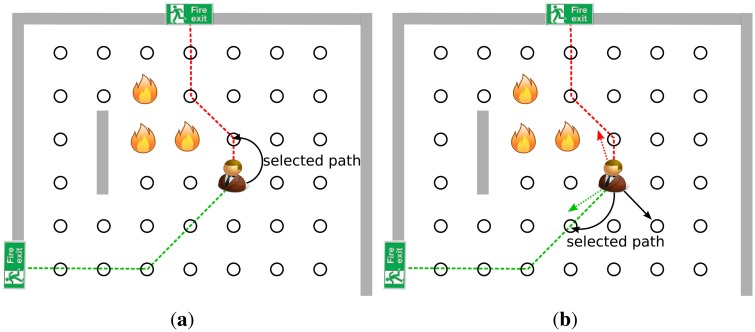
An evacuation example. **(a)** Based on the shortest path the civilian will choose the “red” path; **(b)** By using directions, our algorithm can guide the civilian through the safer “green” path.

Our observations are depicted in Figure 1. This figure shows an example for a fire evacuation with two fire exits and one civilian. As it could be seen, there are two different evacuation path, one for each exit. In the Figure 1a subfigure the civilian will choose the path which is indicated by red, as it is shorter than the other one. However, the “red” path is shorter than the “green” one, but it leads the civilian towards the hazardous area which can be very dangerous. Thus, our idea was that the algorithm should lead the civilian through a safe path even if it is longer than the others. Hence in this case, the civilian should choose the “green” path instead of the “red” one. To predict the safety metric of a path, we can utilize directions, which is the main element of our novel path finding algorithm. A direction vector should be determined for each path, which describes its main direction (the direction points from the civilian position to the selected exit), and another vector should be calculated, which is the optimal evacuation direction from the hazardous area. These direction vectors are shown in Figure 1b. The proposed algorithm uses these directions to guide the civilian through a safe path to a fire exit.

The first step in our algorithm is the calculation of these directions. The directions from the civilian's position to the exits (such as the dashed arrows in Figure 1b) are easy to calculate, since the graph for the given area is stored on the CN, therefore, the positions of the exits are available for every civilian, and each CN can obtain its location from the fixed sensor nodes (SNs). The fire direction (which points from the estimated fire location to the CN) is a bit more sophisticated, since a CN could have many emergency messages (EMs) and these could contain different locations. Thus, the resultant vector should take into account all the received packets. More recent messages (with a newer timestamp) are more relevant, therefore, the locations in the messages should be weighted by factoring in their creation time. Lets assume, that a civilian has received *n* different EMs (identified by (device ID, timestamp) pair), which are ordered chronologically (based on the creation time), and let $\upsilon_{t_{i}}$ be a direction carried by the EM (from the affected location to the current civilian's position) generated at *t_i_*. Then the resultant vector could be calculated as follows:

(1) $$\upsilon_{\text{sum}}^{\to }=\sum_{i=t_{0}}^{tn}\frac{1}{2^{i}}{\vec{\upsilon }}_{i}$$

It should be noted in Equation (1), that when *n* → ∞ then the newest message has the same weight as all the preceding weights aggregated. It is important to notice that the υ*_ti_* vectors are direction vectors, therefore, the result does not depend on the distance between the civilian and the hazardous area.

In the next step, our algorithm tries to find a path based on the previously calculated “fire” vector. Since a civilian has one “fire” vector and many “exit” vectors, the angle between a fire vector and an exit vector can be calculated simply. As it is depicted in Figure 1b the paths with smaller angle (meaning the angle between the selected exit and the fire vector) are more likely to be safer than the paths with higher ones. Hence, a novel cost function could be defined as follows:

(2) $$\hat{F}(\phi_{i},G)=\frac{-\text{cos}(\phi_{i})+1}{2}*D_{i}$$

**Figure 2.**
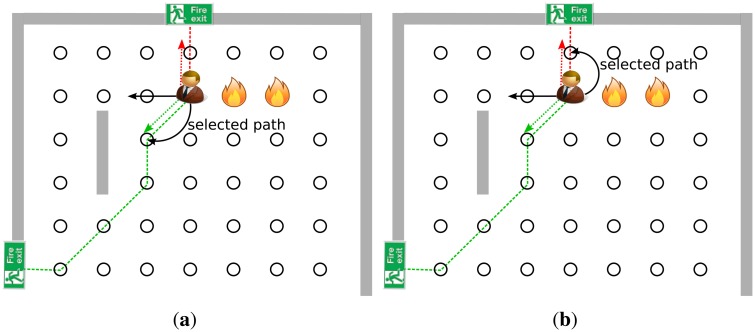
An evacuation where Equation (2) does find a suboptimal path. **(a)** An example where the Equation (2) does not find the optimal path; **(b)** And with the uncertainty factor (Equation (3)).

*φ_i_* denotes the angle between the fire vector and the *i^th^* exit vector, *D_i_* is the shortest path cost to the *i^th^* exit and *G* is the graph of the area. Note, that If there is an exit right next to the civilian's position but its angle (*φ_i_*) is much bigger than the other's (which is actually far from the source), the algorithm will choose the further exit. An example for this case is depicted in Figure 2a. To prevent this, the cost function should be modified with a new factor:

(3) $$\rho_{i}=\frac{\Delta_{i}}{min_{i}D_{i}}*min_{i}{\hat{F}}_{i}$$

*ρ_i_* is called uncertainty factor, and its purpose is to reduce the probability of choosing a suboptimal path based on the directions. Δ*_i_* is the difference between the shortest path's cost to the *i*th exit and the minimum of the shortest path's costs to each exit (Δ*_i_* = *D_i_* − *min_i_D_i_*). Thus, the final version of the cost function is the sum of the previous cost function and the uncertainty factor.

(4) $$F(\phi_{i},G)={\hat{F}}_{i}+p_{i}=\frac{(-\text{cos}(\phi_{i})+1)*D_{i}^{2}}{2*min_{i}D_{i}}$$

Our novel path finding algorithm uses the previously presented cost function (Equation (4)) to select the most appropriate evacuation path. If a CN receives a new EM, it recalculates the direction from the hazardous area, and based on this vector it updates the cost of each path. This method ensures, that the civilian will be guided toward the most appropriate fire exit.

### 2.2. Directional Extension of Epidemic Routing

Most data forwarding and routing protocols for opportunistic networks (oppnets) try to achieve a balance between message delivery ratio and resource consumption [16,17]. For the dissemination of EMs, which are very short in nature, a high delivery ratio and low message latencies are crucial. A fine example of opportunistic communication protocols is the epidemic routing [18], which disseminates multiple copies of a message over the network similar to the spread of an infectious disease used in previous work [19]. The main idea behind the use of the Epidemic Routing protocol was that each EM is intended for all CNs in the system.

In our case it would be beneficial to somehow limit the number of disseminated messages. CNs are not interested in all EMs, only the ones that can change the evacuation path. An EM can change the path due to two reasons; (a) it updates the weights in the graph (*D_i_* will be modified in the cost function) or (b) it changes the previously described fire vector in Equation (1) (*φ_i_* will be modified in the cost function). Since the proposed path finding algorithm uses directions to select the evacuation path (and not the shortest path), and it guides the civilian through a safer path (not through the shortest one), therefore the effect of (a) is less important than (b).

The determination of the fire vector in Equation (1) contains all the received messages in chronological order, thus a threshold (*α_t_*) can be determined whether a newly received message can significantly modify the resultant vector or not. This threshold depends on the location of the exit, the layout of the area and other factors. Therefore, an appropriate threshold should be given for each scenario individually. The older a message is, it represents less weight, so only messages created after the timestamp of the currently received latest message should be taken into account.

The presented novel protocol uses the same transmission mechanism as the Epidemic Routing protocol, but only those messages will be circulated among the nodes, which (a) are created later than the receiver's latest message timestamp; and (b) which determine a new direction (if ${\vec{\upsilon }}_{\text{sum}}*{\vec{\upsilon }}_{sum^{\prime }}<\cos(\alpha_{t}))$ for the evacuation path.

## 3. Performance through Simulation

Although mathematical models [21] can be used to evaluate the performance of subsystems such as for instance the oppcomms, our objective is to evaluate the effect of the proposed directional protocol in the context of a realistic evacuation environment. Thus we have chosen to conduct a simulation study using the DBES tool [20] in a realistic evacuation scenario. In order to compare with earlier results, we use the same parameter values as in [19], with some exceptions.

In our settings, each CN can store 100 EMs for communication purposes, its data transfer rate is 100 Kbits/s and the maximum effective CN communication range is 6 m, since it can be varied between 2–10 m and we have selected a middle range. An SN is located at each vertex of the building graph and each SN uses its own location identifier to locate the CNs. Location messages (LM) are transferred between CNs and SNs, and each LM contains the SN's location identifier. Thus, the navigation system operating on the CNs can determine its current position from the received LMs.

**Figure 3.**
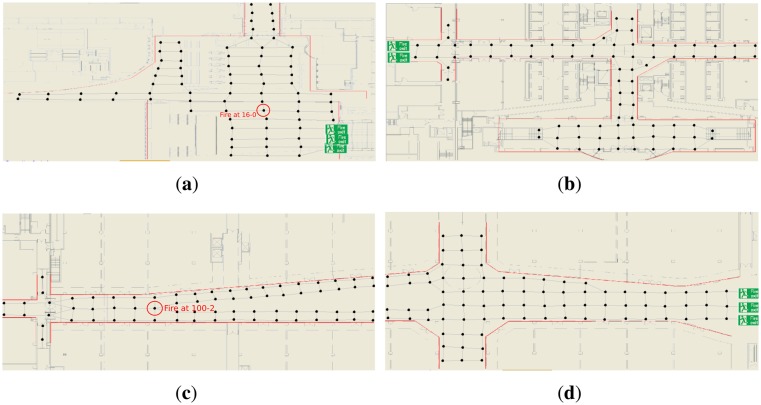
A single-story building used in simulation. The layout parts are connected to each other through the hallways. The string positions of the fire could be seen at **(a)** and **(c)** subfigure. **(a)** The first part of the layout; **(b)** The first second of the layout; **(c)** The third part of the layout; **(d)** The fourth part of the layout.

The simulations are based on a single-story building representation of Figure 3 that was created from the blueprint of a real shopping centre in London. The layout contains 8 fire exits and 321 other nodes, where each node (*i.e.*, graph vertex) has a maximum capacity (in our case it is set to 10). Thus, when a graph vertex has more evacuees than its maximum capacity, that node becomes overcrowded and the simulations take physical congestion into account.

Two different mobility models are used in the simulations. In emergency cases the evacuees move to the selected exit on the shortest path. However, during normal operation the civilians use the following pattern. After waiting for some time which represents shopping or looking around in the store, the civilian chooses a random destination within the building, which is not an emergency exit. After moving to that location (the shortest path between two locations in the building is used) and then waiting for a random time at this destination, it chooses the next destination and so on in a repetitive Markovian random scheme. The average of the random waiting time in our simulations is 10 min. The movement velocity for each civilian is 1 m/s (since a majority of the people in a shopping centre wander from store to store), and we assume that the current health of the civilian does not affect its speed.

Fire hazard with the same intensity is used in all the simulations to achieve a consistent evaluation. The fire spreads along graph edges, following a Bernoulli trial model. The fire model in the simulation and its effects on evacuee health have been inspired by [22]. In the simulations, the fire starts 600 s after the simulation begins and the results are an aggregate of 20 simulation runs with different distributions of evacuees. In all our simulations we did not assume that there was a central alarm in the building to represent the full potential of the self-organized network (formed by the CNs).

Four different simulation scenarios have been tested:

- The emergency system identical to that of [19] for comparison purposes;
- The ESS enhanced with our novel algorithm, where the SNs can determine positions without error (an ideal case);
- The previous system, but operating with positions with errors having a Gaussian distribution with a maximum error of 2 m; and
- The same as the previous one, but with Gaussian errors and a maximum error of 10 m.

### 3.1. Simulation Results

The simulations are performed with four different civilian densities (400, 600, 800 and 1000 civilians) because the number of customers in a shopping centre may vary in a wide range (depending on the day, offers, holidays, *etc.*). Note that 400 civilians on this layout is a sparse presence while 1000 people would be fairly crowded while 600 or 800 could be intermediate cases).

Another important parameter is the starting position of the fire hazard: we have chosen two different locations shown in Figure 3 to start the fire, the first at node 16 − 0 is a critical location as it is very close to 3 exits, so the fire will spread very quickly towards the exits and then these exits become unreachable. The second starting position is less crucial, since the fire will be generated in the middle of a hallway, thus, the civilians can easily escape in different directions.

Many studies of sensor networks for emergency management focus on the technical aspects of the sensor network itself, including the communication system efficiency, the packet travel delays, and the security [23,24]. Here we have taken a different approach, and we essentially focus on the *success* of the evacuation procedure by measuring the state of the evacuees themselves.

**Figure 4.**
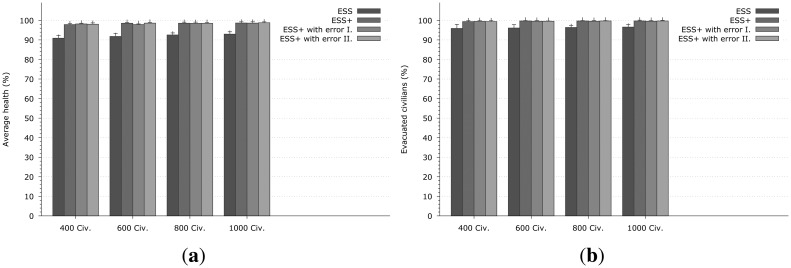
Results of the evacuation when the fire was generated at node 16 − 0. In each group the first column Emergency Support System (ESS) indicates the same emergency system as proposed in [19]. The second one (ESS+) is the enhanced system but without any localization error (an ideal case). The third and fourth are the enhanced version as well but with localization noise (which follows the normal distribution with maximum of 2 and 10 m). **(a)** Average evacuee health *vs.* number of the civilians; **(b)** Evacuated civilians *vs.* number of the civilians.

Simulation results will be shown regarding two different aspects:

- The first, shown in Figure 4, is the main focus of this paper, and it relates to the ultimate success of the evacuation. We measure how many evacuees reach the exit in how much time during the evacuation, and also evaluate their health level when they do exit the system. The percentage indicated is a measure of the effectiveness of the emergency evacuation system, and it captures the primary security metric of the proposed system;
- The second aspect focuses on the mobile self-organized network, and it measures the ratio of messages that are successfully delivered between CNs. This is a performance indicator of the communication protocol that is being used.

The results from the first aspect highlight the differences between the path finding algorithms, while the second set of results focus on the success of the communication protocol.

Figure 4 presents the results from the first aspect when the fire was generated at node 16 — 0. The first column in each group (indicated with ESS label) represents the same emergency system as in the previous work from our group, where the Dijkstra shortest path algorithm is used for selecting the evacuation path and the original version of Epidemic Routing is implemented. When observing the number of evacuated civilians (depicted in Figure 4b) it could be seen that this solution achieves around 95%–96%, which means that in the most dense case 950–960 civilians can be evacuated, but unfortunately the other 40–50 were unable to reach the exits in time. Figure 4a shows the health level (expressed as a percentage) of the successfully exited evacuees, while if a civilian could not exit the building, his/her health is not taken into account). The evacuated civilians have around 90%–93% of their health with the ESS. One important thing should be noticed, namely that the ESS (and other systems as well) gives better results when the density of the civilians is higher (so the performance of the ESS is dependent on population density). This is the positive impact of the self-organized mobile networks formed by the CNs: more nodes mean better communication opportunities therefore EMs can be delivered faster and more reliably to all CNs.

The second, third and fourth columns show results for the enhanced system that uses our algorithm with the error model being used for the localization. ESS+ is an ideal scenario with no error in the positioning system, therefore, each SN can tell the exact position of a CN. Obviously this is not a real-life assumption. We also show a normal distribution with the maximum error set to 2 m (I) while in (II) it is 10 m. The performance of ESS+, can achieve about 3%–4% better results on evacuated civilians and 6%–7% on the health of the civilians than just simply ESS. The 3%–4% improvement in the number of the evacuees means 30–40 people in the overcrowded scenario. The proposed algorithm does not only rescue more people from the building, but the evacuated civilians have better health as well (Figure 4a).

These results indicate that the directional based path finding algorithm can calculate the appropriate evacuation path with or without positioning error, since the variants of ESS+ achieve nearly the same results with differences well within statistical variations due to the simulations, and the proposed path finding algorithm appears to be tolerant to positioning errors.

**Figure 5.**
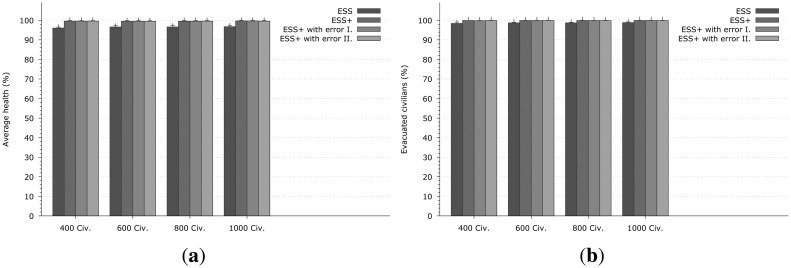
Results of the evacuation when the fire was generated at node 100 − 2. **(a)** Average evacuee health *vs.* number of civilians; **(b)** Evacuated civilians *vs.* number of civilians.

Other simulation results obtained when the fire starts at node 100 − 2 are shown in Figure 5. The same conclusions can be drawn as from Figure 4, while in this case the differences between the enhanced and the normal version of ESS are smaller with regard to the number of successful evacuees in Figure 5b that improves by 2%, and by 3% with regard to health in Figure 5a, though this improvement is not negligible since 2% of the evacuees is 8–20 rescued people.

The lower difference is because of the starting location of the fire hazard. This location (as it is depicted in Figure 3) is in the middle of a long hallway with no fire exits. In this case the shortest evacuation path to the nearest exit could be a good (but definitely not the best) solution as well, since the evacuation path from the hazardous area to the nearest exit is much less likely on the path of the fire, because the fire is being spread from a centre node towards the exits.

**Figure 6.**
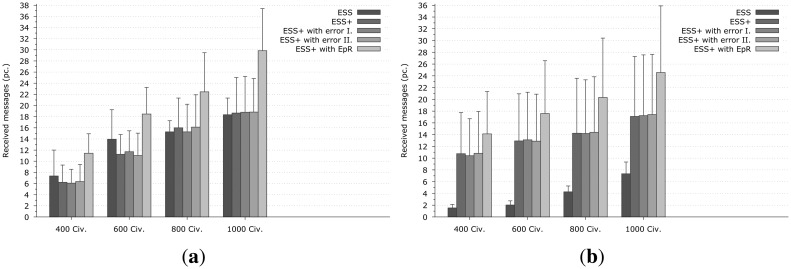
Communication results of the emergency systems. The starting position of the fire was node 16 − 0 at **(a)** and 100 − 2 at **(b).** The first four columns in each group is the same as in previous figures, however, the fifth is a new one, indicating the enhanced ESS with Epidemic Routing. **(a)** The fire was generated at node 16 − 0; **(b)** The fire was generated at node 100 − 2.

Our final results relate to resource consumption in the mobile self-organized network formed by the CNs. Figure 6 shows the average number of the received EMs in a CN. The figure contains 5 columns instead of 4 in each group, since the fifth is the ESS+ system but implemented with the Epidemic Routing protocol instead of our communication protocol. This is essential to draw the right conclusions as nodes with ESS are forming different networks than with ESS+. To compare the two protocols, the same networks and the same conditions should be established (so in our aspect the first column here is meaningless, it is shown only for completeness). As it could be observed, the extended version of the Epidemic Routing protocol can achieve much better results, than the original version, when the number of duplications is taken into account. This is because Epidemic Routing protocol transfers all the new messages between the communication nodes, while the novel version transfers only those which fulfill two requirements. The first one is that the timestamp of the EM must be more recent than the receiver node's latest EM, and the second is that the contained direction must enclose a bigger angle with the resultant direction vector then *α_t_*. In our simulations the *α_t_* has been chosen to *π*/4. Obviously it can be seen as well, that increasing the density of the civilians, the number of the transferred EMs is increasing, since the average size of the network formed by the CNs is bigger as well. The second, third and the fourth columns (which indicate the ESS+ system) show almost identical results, so it can be stated, that the extended version of the Epidemic Routing is fault-tolerant as well.

## 4. Conclusions

We have proposed and described a novel path finding algorithm and communication protocol to enhance autonomous ESSs. This path finding algorithm exploits the location of the communication nodes and hazards to calculate the direction from the hazardous area to the evacuee's current location to search for a safe path to a nearby exit. The communication protocol, is an extension of Epidemic Routing.

A real-life example of a shopping centre was used as a case study to evaluate how these two algorithms can improve the performance of emergency evacuation systems. We evaluated the performance based on four different densities evacuees and with two different fire starting positions. Our experimental results indicate that an emergency evacuation system enhanced with our novel algorithms can evacuate a few percentage more evacuees and also improve the health level of the evacuees. These can lead to a significant number of people who are actually saved during an emergency such as a fire in a crowded shopping centre. In future work it will be quite interesting to evaluate the inclusion of more sophisticated guidance techniques that could include real-time visual matching with enhanced reality tools [25], in order to improve the decision making and provide evacuees with better and more reliable directions that take into account both the evacuees precise physical context and his/her location.
